# Photo-Mechanical Response Dynamics of Liquid Crystal Elastomer Linear Actuators

**DOI:** 10.3390/ma13132933

**Published:** 2020-06-30

**Authors:** Przemysław Grabowski, Jakub Haberko, Piotr Wasylczyk

**Affiliations:** 1 Photonic Nanostructure Facility, Faculty of Physics, University of Warsaw, ul. Pasteura 5, 02-093 Warsaw, Poland; pgrabowski@fuw.edu.pl; 2 Faculty of Physics and Applied Computer Science, AGH University of Science and Technology, Al Mickiewicza 30, 30-059 Kraków, Poland; haberko@fis.agh.edu.pl

**Keywords:** liquid crystal elastomer, photo-mechanics, light-powered actuator, micro-robotics, LCE degradation

## Abstract

With continuous miniaturization of many technologies, robotics seems to be lagging behind. While the semiconductor technologies operate confidently at the nanometer scale and micro-mechanics of simple structures (MEMS) in micrometers, autonomous devices are struggling to break the centimeter barrier and have hardly colonized smaller scales. One way towards miniaturization of robots involves remotely powered, light-driven soft mechanisms based on photo-responsive materials, such as liquid crystal elastomers (LCEs). While several simple devices have been demonstrated with contracting, bending, twisting, or other, more complex LCE actuators, only their simple behavior in response to light has been studied. Here we characterize the photo-mechanical response of a linear light-driven LCE actuator by measuring its response to laser beams with varying power, pulse duration, pulse energy, and the energy spatial distribution. Light absorption decrease in the actuator over time is also measured. These results are at the foundation of further development of soft, light-driven miniature mechanisms and micro-robots.

## 1. Introduction

Scaling down mechanical devices, in particular robots, calls for new approaches to power sources, motors, drivetrains, and actuators. In the millimeter scale and below, traditional battery-powered electric motors and gears fail to deliver, mainly due to problems with fabrication of multiple-part assemblies and on-board power supply.

As an alternative, soft actuators, driven with different stimuli, have been demonstrated in small scales [1]. Dielectric elastomer actuators (DEAs) are based on electrostatic interaction between flexible electrodes with a compressible membrane sandwiched in between [2]; large strains are possible here, but very high voltages are required [3]. Conjugated, conductive polymers have also been used to make electrically driven actuators [4]. Asymmetric swelling of gels in ionic solutions can generate shape changes too [5]. A large class of magnetically driven actuators have been developed, mostly based on soft polymers with suspensions of magnetic particles and different modes of actuation are possible via temporally and/or spatially varying external magnetic fields [6,7]. Small scale actuators can also be powered by chemical reactions that induce the material deformation, by e.g., selective modification of chemical bonds in polymers [8]. Pressure driven soft actuators rely on structures with spatially varying stiffness that deform upon expansion of gas- or liquid-filled chambers [9]—they have been realized on a small scale [10], but still need the tubing to deliver the pressurized medium.

**Figure 1:**
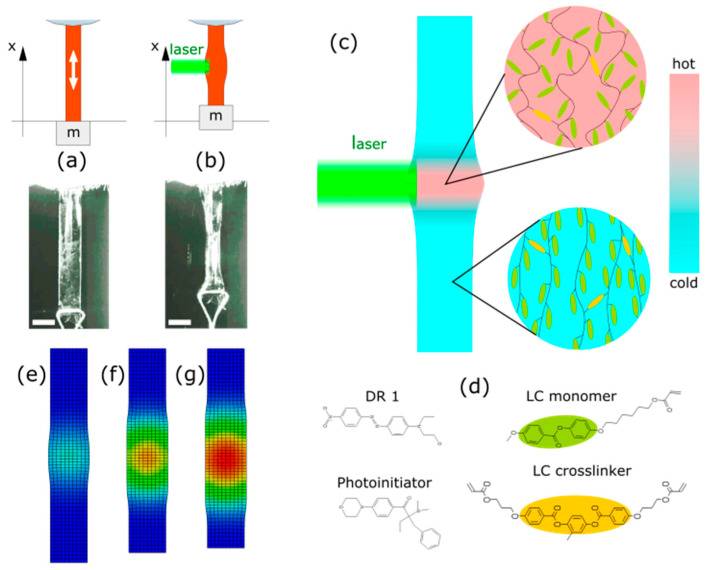
Linear light-driven actuator made of liquid crystal elastomer (LCE) film. A strip cut from an LCE film with nematic orientation is suspended with a weight at the lower end. When illuminated with a laser beam, it contracts along the orientation of the director (shown with the white arrow) due to photo-mechanical effect in the elastomer–schematic and photographs of the experimental setup with the relaxed (light off) (**a**) and actuated (light on) (**b**) strip. The visible narrowing of the central part of the strip in the photographs is due to curling caused by the residual stress in the LCE film. Absorbed light energy locally increases the elastomer temperature and the order⇆disorder transition occurs, resulting in a macroscopic deformation (contraction) (**c**). Molecules used in the LCE composition: liquid crystal monomer, crosslinker, photoinitiator triggering the polymerization upon UV light absorption and the red dye (Disperse Red 1) (**d**). The colors in the molecular structures correspond to these in (**c**). Snapshots from the finite element method numerical simulations of the LCE actuator deforming upon illumination with a Gaussian laser beam (**e**–**g**). The colors scale indicates the elastomer temperature. The white scale bars on the photographs are 2 mm long.

Light induced actuation has the advantage of remote power transmission and fast, local control from centimeter to micrometer scales [11]. “Pure optical” response, based on reversible photoisomerization of azobenzene molecules in polymers or gels, typically requires two wavelengths and has response time on the order of seconds [12]. Photo-thermal actuation is realized via heating, with light absorption from e.g., a laser beam, followed by (anisotropic) thermal expansion [13] or phase transition [14] (Figure 1a–c); it can use one light color and be very fast, depending on the actuator size [11].

Liquid crystalline elastomers (LCEs) are solid, soft polymers with well-defined alignment of molecules, characteristic for the liquid crystals. Their rod-shaped molecules are aligned in cross-linked polymer chains and, due to this arrangement, the order(anisotropic) ⇆ disorder(isotropic) transition reduces the effective chain length along the alignment direction (the director), at the same time, increasing the spacing between the molecules in the perpendicular directions. Consequently, the material can exhibit large, fast and reversible shape changes upon external stimulus, e.g., light absorption [15] (Figure 1c). The exact topology of this macroscopic deformation is determined by the orientation of the director in the elastomer element volume, its shape, and the stimulus spatio-temporal distribution. The mechanisms responsible for the photo-mechanical response in general fall into two categories: it can be either photochemical reactions, often associated with the cis-trans isomerization [16,17], or photo-thermal heating, when the temperature of the material (locally) increases due to light absorption in a dye [18,19]. Liquid-crystal elastomer elements can be fabricated in the scales from centimeters (10–100 µm thick films, made in glass cells) to micrometers (with one- or two-photon laser photolithography [20]). Simple light-powered actuators can contract, bend, and twist, and more complex modes of deformations are also possible [21,22,23]. Engineering the molecular alignment led to demonstration of a 5 mm diameter rotary motor that uses a travelling deformation in a ring-shaped rotor, frictionally coupled to the stator—similar to the piezo motors used in autofocus camera lenses [24].

LCE actuators are among the promising candidates for actuators in small scale mechanics and robotics [25]—they can be remotely controlled with spatially and/or temporarily modulated light beams and, due to their soft nature and elasticity, can be used directly to realize various concepts of soft, untethered robots [26]. Furthermore, the material itself can be programmed to respond differently in different situations, e.g., selectively interacting with objects having certain optical properties [27]. Light-powered LCE elements have been used so far with various success as micro actuators [28,29,30] and in small scale soft robots [31].

To progress towards reliable small-scale mechanics and micro robotics based on photo-responsive materials, a better understanding of the light-driven actuator dynamics is necessary. In this paper, we experimentally examine various aspects of an LCE linear actuator operation: the response (stroke) vs. light power upon continuous and pulsed illumination as well as the response dynamics (transient behavior). We look into the interplay of the energy influx and heat dissipation with different light pulse durations and the influence of the light spatial energy distribution. These results are accompanied by a finite element method (FEM) modeling of the linear actuator response. Degradation of the photo-responsive molecules is often neglected in the context of photo-responsive materials—we take a closer look at the absorption evolution of the elastomer actuator with the accumulation of the absorbed light energy.

## 2. Materials and Methods 

### 2.1. Liquid Crystal Elastomer Film Fabrication

The liquid crystal polymer mixture consisted of commercially available compounds: 78 wt % of the LC monomer (Synthon, Bitterfeld-Wolfen, Germany), 20 wt% of the LC crosslinker (Synthon), 1 wt% of the photoinitiator (Irgacure 369, Sigma Aldrich, St. Louis, MO, USA) and 1 wt% of the dye (Disperse Red 1, Sigma Aldrich), Figure 1d. All compounds were used as received. The mixture was melted and stirred in a glass flask at 80 °C on a hot plate with a magnetic stirrer.

The glass cell was made of two microscope slides with 10 μm glass beads used as spacers. The nematic orientation of the molecules in the cell was induced by polyvinyl alcohol (PVA) alignment layers spin-coated on both slides (10% w/w PVA in water, 6000 rotation per minute (RPM) for 60 s). After spin coating, the slides were dried at 80 °C for 5 min. Mechanical rubbing was performed with a home-built rotating drum rubbing machine (YA-19R rubbing fabric). For fabrication of the LCE film with uniform nematic orientation, the glass slides were aligned with the rubbing direction parallel at the top and the bottom slide.

The cell was filled with the molten LC mixture by capillary forces at 80 °C and was passively cooled down to 40 °C, so that the molecules aligned. During cooling, the LC domains grew and merged together.

High power 379 nm LEDs were used for the film polymerization, with the simultaneous illumination for 20 min from both sides of the cell. After polymerization, the cell was dipped in water at room temperature, wedged open, and the LCE film was removed from the cell. The 18 × 2 mm strip was cut with a razor blade, with the longer side parallel to the director (molecular orientation). The molecular alignment in the film was verified with light transmission from a white light source and crossed polarizers.

### 2.2. The Linear Actuator Response Measurements

The LCE strip was hung with one end clamped and weighed down with a 0.33 g mass at the free end (Figure 1a,b). This small mass was used to keep the strip straight, as there may be other unwanted deformations, like bending or twisting, resulting from e.g., the residual stress in the LCE film introduced during fabrication (cooling). A CMOS digital camera (1280 × 720, 6 fps or 1280 × 960, 27 fps) was used to record the position of the loose end—the actuator stroke. Each frame used for measurement was extracted as a 1280 × 720 PNG file with OpenShot Video Editor (2.4.4). The images were processed using Fiji ImageJ freeware (2.0.0-rc69/1.52p) [32].

A CW 532 nm laser (Verdi V-5, Coherent, Santa Clara, CA, USA, 5.0 W maximum power, 3.0 mm Gaussian beam full width at half maximum (FWHM) measured at the LCE strip surface) was used for actuation. A fast-switched galvo scanner mirror was used to steer the beam onto the LCE actuator for a controlled period of time. To produce laser beams with different cross sections, a cylindrical lens was used. To measure the LCE strip contraction (the actuator stroke), it was filmed at an angle along the laser beam (Figure 1b) with a digital camera installed on a microscope and position of a marker at the strip end was determined on the movie frames.

The LCE film absorption degradation was determined by measuring light transmittance through the LCE strip. The central part of the film strip was irradiated with pulses of green light of increasing intensity (3.0 mm gaussian beam FWHM, 0.63 W for 65 s, 1.27 W for 50 s, 1,91 W for 10 s, and 3.19 W for 178 s). After each pulse, a low-power (4.4 mW) green beam was sent through the irradiated area and the transmitted power was measured behind the film to determine its transmittance.

### 2.3. Numerical Simulations of the Photo-Thermal Deformation

The photo-mechanical response of the LCE actuator was simulated with the finite-element method (FEM) using a coupled temperature-displacement analysis in the Abaqus software (Abaqus 6.13, Dassault Systèmes, Vélizy-Villacoublay, France). Relatively low total contraction of the material allowed us to treat it as a linear elastic material, without introducing hyperelastic properties and nonlinear constitutive equation. In the simulations the contracting LCE actuator was a 12 mm × 2 mm × 10 μm cuboid made of an orthotropic material, with a negative temperature expansion coefficient α_33_ = −0.005 along the initial director orientation and positive expansion coefficients α_11_ = α_22_ = 0.0025 in two orthogonal directions. The material volume upon contraction was preserved (Figure. 1e-g). We assumed the LCE strip undergoes the shape change within the 80–120 °C temperature range, and the total contraction along the director in this range is 20% [11]. The laser stimulus was a body heat flux with a spatial Gaussian power distribution on the LCE actuator surface (FWHM = 3 mm) and controlled duration, to match the conditions of the experiments and the 10 μm-thick LCE film absorbed 60% of the incident power. As the LCE film was very thin in comparison to its other dimensions, only one finite element was used in the computer model in the direction perpendicular to the surface.

**Figure 2:**
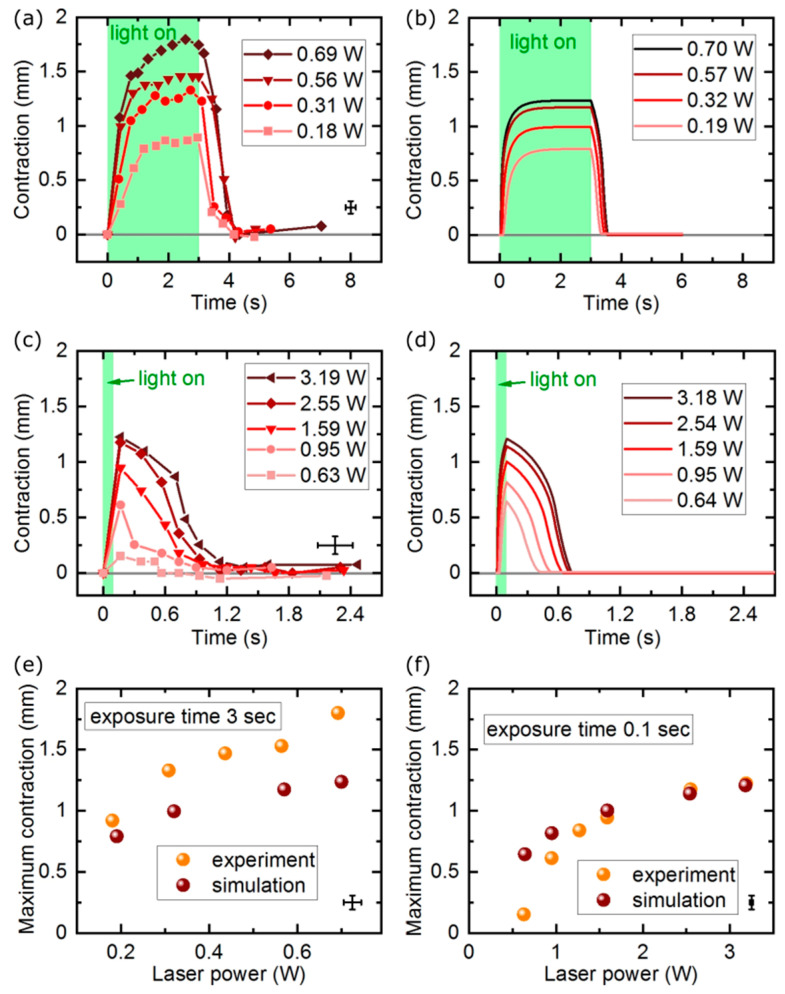
Linear actuator response with continuous and pulsed illumination. Measured and simulated actuator contraction vs. light power with long (CW) light exposure. (**a**,**b**) measured and simulated actuator contraction vs. light power with short (pulsed) light exposure; (**c**,**d**) measured and simulated maximum stroke for the CW illumination **(e)** and pulsed illumination (**f**).

**Figure 3:**
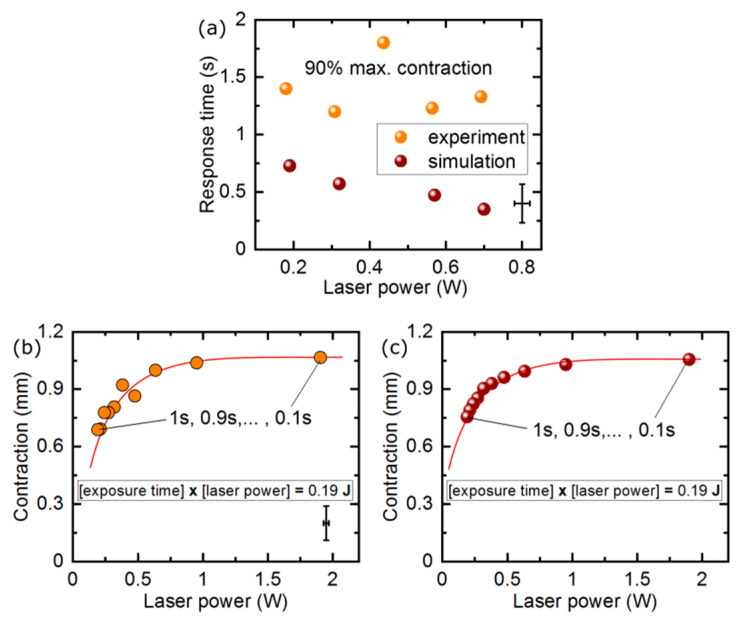
Linear actuator response time and stroke for different laser pulse duration. The actuator response time from zero to 90% of the maximum contraction retrieved from the measurement data in Figure 2a and simulation data in Figure 2b (**a**). Measured and simulated actuator response with the same amount of light energy delivered via laser pulses with varying duration-measurement (**b**) and simulations (**c**). The red lines are guides for an eye only.

**Figure 4:**
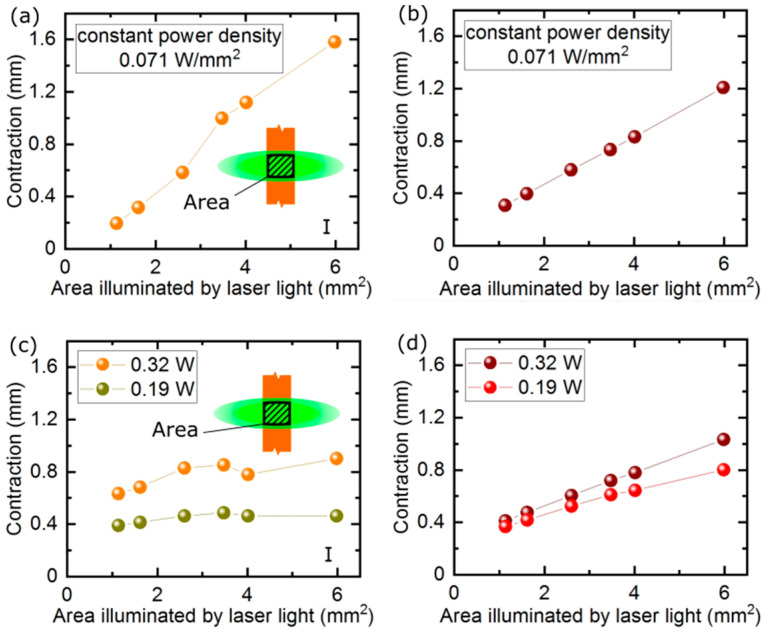
Linear actuator response with different spatial distribution of the light energy in the laser beam. Measured (**a**) and simulated (**b**) actuator response upon illumination with laser beams of increasing size and constant power density. Measured (**c**) and simulated (**d**) actuator response upon illumination with laser beams of constant power and varying size.

Other material properties were set to the values of the high-density polyethylene: mass density 960 kg/m^3^, thermal conductivity 0.49 W/m∙K, Young’s modulus 1.035 GPa and Poisson’s ratio 0.4. In the course of the simulations, we verified that the exact values of these parameters did not considerably influence the photo-mechanical response of the actuator. The heat capacity was determined using an optimization procedure in which its value providing the best agreement with the experimental results was found: 1200 J/kg∙K, a reasonable value for a polymer material. The actuator exchanged heat with the environment (air at 24.5 °C) via radiation (emissivity ε = 0.9, also a value typical for polymers) and convection (a moderate heat transfer coefficient of 20 W/m^2^⋅K was found after a series of optimization steps). The FEM simulation results are presented along the experimental data in Figure 2, Figure 3 and Figure 4 and Video S1 (Supplementary Materials). There is overall qualitative, and in some cases also quantitative, agreement between the experiments and simulations.

Performing multiple simulation runs with different parameters gave us insights into the contributions of individual factors to the photo-mechanical dynamics of the LCE actuator. Convective heat transfer coefficient *h* has a very strong impact on the maximum contraction (stroke)—increasing *h* decreases the maximum stroke, as heat is more efficiently released from the system, leading to lower average temperature. However, a high value of *h* also leads to larger differences between maximum contraction when high and low laser powers are used. Increasing the LCE heat capacity *Q* results in lower maximum stroke, as more energy needs to be delivered into the material to increase its temperature. On the other hand, the value of thermal conductivity *k* has very little impact on the actuator response dynamics—as the LCE film is only 10 μm thick, little energy is transported across the strip surface.

## 3. Results and Discussion

In the first experiment, we measured the actuator response (stroke on contraction) to illumination with a laser beam with constant spatial profile and varying power. These measurements were performed in two regimes: (1) stationary, when the light energy was delivered for long enough time, so that the actuator reached the steady state, and (2) impulsive, when the light energy was delivered in pulses shorter than the transient response time of the actuator and it did not reach the stationary state.

In the basic configuration, the actuator was illuminated with light that was absorbed efficiently by the dye—for the green 532 nm doubled Nd:YAG laser, the DR 1 red dye was chosen that has the peak absorption near this wavelength—and the photo-mechanical response (contraction) was a function of the light power. In the stationary state, the light energy that was absorbed and transformed into heat, and the thermal energy released from the LCE element through thermal conductivity, convection, and radiation in a unit time, were equal, the actuator reached thermal equilibrium and, after some time, the contraction was constant over time (Figure 2a,b).

Within the laser power range below the elastomer damage threshold, the actuator stroke in both the continuous and pulsed illumination regimes increased with the increasing laser power.

Measured response time of our LCE actuator was 1.39 ± 0.28 s and was independent of the laser power (Figure 3a). Assuming a simple shape (cube, cylinder, strip of film), the photo-mechanical actuator response time depends on the actuator characteristic size. The light absorption geometrical cross section that determines the amount of energy absorbed and the surface area in contact with the environment that is responsible for the heat exchange scale quadratically with size. At the same time, the actuator volume (and mass) to be heated scales cubically with size. As a result, the response time of light-driven actuators based on thermal effects decreases with their size and was measured to be as small as a few milliseconds for LCE structures with dimensions on the order of tens of micrometers, e.g. those fabricated with laser photolithography [11].

For application when a fast actuator response is used, the LCE elements may work in the regime when their photo-mechanical response does not saturate. Light energy is delivered in a shorter time than the elastomer actuator response time. In the next experiment, the actuator stroke was measured upon illumination with 100 ms pulsed laser beam of different power. Even though the maximum contraction was not reached, the actuator produced a significant stroke that depended on the total light energy delivered (Figure 2c–f) and was beginning to saturate at the maximum laser powers used.

When experimenting with pulsed illumination of the light-responsive actuators, an interesting question arises: how does the photo-mechanical response compare for a long laser pulse with low instantaneous power and a short pulse of higher instantaneous power, both delivering the same energy to the light-responsive element? In Figure 3b, we present the measured maximum actuator stroke for pulse durations between 1 s and 100 ms, with the laser power adjusted so that the total energy in each laser pulse was always 0.19 J. For long pulses, the actuator response was visibly smaller—in these conditions, the time constants governing the heat exchange between the LCE strip and the surrounding air allowed for the effective cooling (likely mainly due to heat conductivity) and the absorbed light energy was dissipated before it could significantly increase the actuator temperature. If, on the contrary, the same energy was delivered in a short pulse, it could heat the material up before other thermal processes came into play and the actuator response was larger. For even shorter pulses, there was no difference in the total stroke within our measurement system resolution, as the final temperature reached was the same.

In many demonstrations of soft, light-driven robots, the LCE element illumination is provided by laser beams that are modulated in space and/or time and illuminate only some fragments of the light-responsive elements. One example is the travelling wave concept [33], used to demonstrate the caterpillar- and snail-like locomotion of millimeter scale soft robots [22,34] and swimming at an even smaller scale [35].

In the first experiment on the beam size dependence of the actuator response, we illuminated the LCE strip with a laser beam of constant power density, but different size. As expected, when the illuminated area increased, the actuator response increased, as larger and larger volume of the elastomer contributes to the total stroke (Figure 4a,b).

A more interesting question is: what is the actuator response if the same amount of light energy is delivered with two laser beams—one of a large cross section, but lower intensity and the other of a smaller cross section, but larger intensity, so that the total power is the same in the two beams? Our next experiment demonstrated that, when the actuator was illuminated with a laser beam of constant power, but of varying size, the photo-mechanical response was independent of the illuminated area (Figure 4c). This indicates that, for a small beam, the contraction of the illuminated volume is proportionally larger, as the power density is higher and, for larger beams, the power density decreases, but a proportionally larger area is illuminated and contributes to the total photo-mechanical response and thus to the net contraction. For the FEM simulations, there is a linear dependence (Figure 4d), indicating that some material parameters may not be approximating the real actuator or some saturation effects come into play in the experiment.

**Figure 5:**
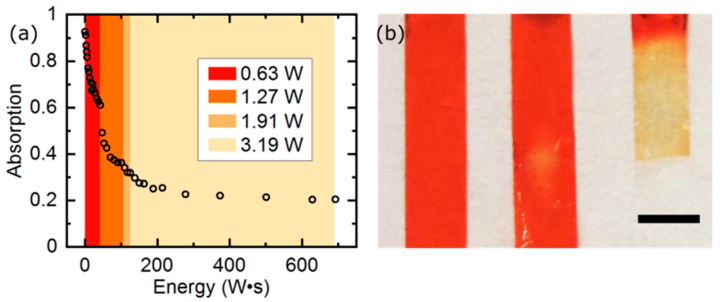
Measured LCE actuator absorption loss vs. the total light energy absorbed. Total absorbed light energy, calculated as the absorbed laser power times the exposure time, is plotted on the horizontal axis. Increasing laser power was used in four consecutive time ranges to speed up the experiment (**a**). Optical photograph of a 10 μm thick LCE film with 1 wt % of DR 1. Before the experiment (0 J total absorbed energy, left) and during the experiment (50 J total absorbed energy, center). Dye degradation is visible as a transparent spot in the center of the laser illuminated area. At the end of the experiment, the entire irradiated area became transparent and the actuator was broken (above 700 J total absorbed energy, right) (**b**). The black scale bar is 2 mm long.

One of the rarely mentioned aspects of using light-responsive smart materials, in particular based on liquid crystal elastomers, is their degradation after exposure to large doses of radiation. To look into this issue, we performed absorption measurements of the LCE film of the same type as used in the linear actuator presented above. The initial absorbance of the film with 1 wt% of the red dye was 93%. After irradiation with pulses of green light, absorption in the illuminated region decreased significantly and, after the total absorbed power reached around 300 J, it saturated at around 20% (Figure 5a). The dye degradation was clearly visible—the film turned transparent, where it was originally deep red (Figure 5b). Assuming a moderate laser power of 0.3 W, one activation per second and 50% actuator duty cycle, the 300 J energy absorbed corresponds to 2000 cycles of contraction, which gives an estimate of the (rather unimpressive) LCE actuators lifespan under such conditions. Instead of dyes, other, more stable light absorbers can be used, such as gold [36], silver, [37] or carbon nanoparticles [38].

## 4. Conclusions

Despite much effort and some spectacular demonstrations of autonomous insect-size robots [39], the technology of autonomous mechanism at and below the millimeter scales is far from mature. If the design of new machines based on photo-responsive elastomers is to go beyond single contracting or bending strips of a stimulus-responsive material, towards more complex modes of operation, better understanding of the photo-mechanical response of LCE actuators is needed. The light-induced deformation of liquid crystal elastomers can be sensitive to the light spectrum [40] or polarization [41]. This offers more degrees of freedom much needed for the realization of the non-reciprocal motion and other advanced functionalities.

Our experiments indicate that: (a) if used in the transient regime, the actuator response time is independent of the laser power, (b) for pulsed laser actuation, long laser pulses result in smaller actuator response, compared to short laser pulses delivering the same energy; this is true down to a certain pulse duration, determined by the time constants of heat transfer to the environment, (c) when part of the actuator is illuminated with a laser beam of constant total power, but of varying size, the photo-mechanical response is independent of the illuminated area. Using dyes may enable color-sensitive light actuation with many degrees of freedom in complex light-driven mechanisms, but the actuator durability may be compromised due to light absorption deterioration with increasing accumulated absorbed energy.

A finite element method (FEM) model, using anisotropic thermal expansion/contraction defined by the director distribution, proved to work well for the light-driven LCE actuator simulations, also with spatially and/or temporally modulated laser beam stimuli and can be used to further understand, test, and design more complex elements with photo-mechanical responses.

While scaling down machines, the laws governing the mechanics of the micro-world come into play—while the gravity or electromagnetic forces are governed by the same constants, their relations change with scale and new challenges as well as new possibilities emerge: crawling on an upside-down glass ceiling is easy for the millimeter scale snail robot [34], while a micron-size walker may struggle to lift its legs, anchored to the ground by van der Walls forces [11].

Light-driven micro-mechanics and micro-robotics are still at their infancy and we are sure to witness many spectacular demonstrations in the years to come.

## Supplementary Materials

The following are available online at https://www.mdpi.com/1996-1944/13/13/2933/s1, Video S1: One of the results of the finite element method (FEM) simulations: the linear LCE actuator contracting with the laser beam on and expanding back with the laser off.
